# Interleukin-13 +2044 G/A and +1923C/T polymorphisms are associated with asthma susceptibility in Asians: A meta-analysis

**DOI:** 10.1097/MD.0000000000009203

**Published:** 2017-12-22

**Authors:** Quanhui Mei, Jingjing Qu

**Affiliations:** a Department of Intensive Care Unit, The First People's Hospital of Changde City, Changde, Hunan; b Department of Medical Oncology, Lung Cancer and Gastrointestinal Unit, Hunan Cancer Hospital, Affiliated Cancer Hospital of Xiangya School of Medicine, Changsha, China.

**Keywords:** +1923C/T polymorphisms, +2044 G/A polymorphisms, Asians, asthma, IL-13

## Abstract

A number of studies have reported that the interleukin 13 (IL-13) gene is associated with asthma susceptibility. However, the reported relationships between the +2044G/A and +1923C/T polymorphisms and asthma susceptibility are inconsistent, especially in Asian adults and children with atopic status. Meta-analysis was used to analyze combined data.

The +2044G/A and +1923C/T polymorphisms were investigated using data from 18 and 11 studies, respectively. The results suggested that there was an association between asthma and the IL-13 +2044G/A polymorphisms: odds ratio (OR) 1.34, 95% confidence interval (CI) 1.03–1.75 for AA versus GG + GA and +1923C/T; OR 1.50, 95% CI 1.26–1.78 for TT versus CC; and OR 1.15, 95% CI 1.10–1.21 for TC versus CC. The subgroup meta-analysis demonstrated that IL-13 +2044G/A polymorphisms are associated with asthma: OR 1.47, 95% CI 1.06–2.04 for AA versus GG + GA and +1923C/T; OR 1.70, 95% CI 1.26–2.30 for TT versus CC; and OR 1.27, 95% CI 1.03–1.56 for TC versus CC. In particular, IL-13 +2044G/A polymorphisms are specifically associated with Asian ethnicity in both adults and children with atopic status. However, the 1923C/T polymorphisms were not significantly associated with age group or atopic status within the Asian subgroups. Further investigation using larger samples and meta-analysis is required. No publication bias was detected.

This meta-analysis indicates that the IL13 +2044G/A and +1923C/T polymorphisms are risk factors for asthma, especially among Asians.

## 1 Introduction

Asthma is a chronic inflammatory disease in which many cells of the innate and adaptive immune systems act together with inflammatory cells to cause bronchial hyper-reactivity (BHR), airway smooth muscle contraction, mucus overproduction, and airway remodeling. Worldwide, more than 300 million people are affected by asthma. The total cost of the disease is estimated to exceed $18 billion annually in the USA, including direct medical costs and indirect costs of lost productivity.^[1]^ The asymmetric functions of T helper cells 1 (Th1) and 2 (Th2) play an important role in causing asthma.^[2,3]^ Mice lacking key Th2 cytokines interleukin (IL)-4, IL-5, or IL-13 have reduced inflammation reactions in asthma features in the ovalbumin model.^[4]^ IL-13 is important for BHR and for goblet cell metaplasia, which can produce thick mucus containing the releasing mucins MUC5AC and MUC5B, which obstruct the airway lumen.^[5,6]^

It is well-known that single-nucleotide polymorphisms (SNP) can be used to assess genetic disorders, on which basis multiple phenotypes have been associated with altered levels of IL-13. Two SNPs, positioned at regions +2044G/A and +1923C/T, have been linked to effective production of IL-13. Heinzmann et al^[7]^ first reported an association between the IL13 G+2044A variant and high asthma risk in Japanese and British populations. Recent studies have suggested that IL-13 +1923C/T polymorphism plays a critical role in the development of asthma. Although +2044G/A polymorphisms have been correlated with asthma susceptibility in several studies,^[8–10]^ neither the influences of age nor Asian populations have been described. One study^[11]^ reported a relationship between +1923C/T polymorphisms and asthma susceptibility; however, the influences of atopic phenotypes and age were not analyzed in Asians. Thus, pooled analysis of all available studies is necessary.

In this study, we sought to determine the association between IL-13 +2044G/A and +1923C/T polymorphisms and asthma susceptibility in Asian specifically, by including recently published studies, to evaluate the pathogenesis of asthma from a novel perspective.

## 2 Materials and methods

### 2.1 Search strategy and data extraction

This meta-analysis was not involved in an ethics committee or institutional review board. We considered all studies that examined the association between IL-13 and asthma, identified using PubMed, EMBASE, and China National Knowledge Infrastructure database (Wanfang database) from 2001 to 2014. Search terms were as follows: (gene or allele or polymorphism) and (IL-13), (+2044G/A), or (+1923C/T and asthma). Searches were performed in duplicate by 2 independent reviewers (QHM and JJQ). Only English and Chinese-language papers were included.

To explain the relationship between IL-13 and asthma susceptibility in Asians, different genotypes of +2044G/A and +1923C/T polymorphisms were extracted. The following information was extracted from each study: author, publication year, country, ethnicity, age group, positions, atopic status, and the number of cases. Disagreements were resolved by discussion between the authors.

### 2.2 Inclusion and exclusion criteria

All selected studies were evaluated for the following inclusion criteria: evaluation of the +2044G/A and +1923C/T polymorphisms in IL-13 gene and asthma risk; inclusion of odds ratio (OR) and 95% confidence interval (CI) estimates; use of a case-control design. Studies were excluded on the basis on the following criteria: not relevant to IL-13 +2044G/A and +1923C/T polymorphism or asthma risk; reviews or abstracts; sample size not reported; and animal study. For overlapping studies, the most recent or largest-sample report was selected.

### 2.3 Assessment of quality scores

The quality of the included studies was evaluated by JJQ using a standardized quality assessment scoring system for studies of genetic association with asthma, as recommended by Thakkinstian et al.^[12]^ The criteria covered the representativeness of cases and controls, ascertainment of cases and controls, genotyping examination, Hardy-Weinberg equilibrium (HWE), association assessment, and response rate. Total scores ranged from 0 (worst) to 15 (best). Any disagreement was adjudicated by a third investigator (JJQ). Low-quality studies with scores of 4 or lower were excluded from analyses.

### 2.4 Statistical analysis

We used the meta-analysis method described by Thakkinstian et al.^[12]^ Briefly, ORs OR1, OR2, and OR3 were employed to calculate the genetic effects: AA versus GG (OR1), AG versus GG (OR2), and AA versus AG (OR3) for +2044G/A; TT versus CC (OR1), TC versus CC (OR2), and TT versus TC (OR3) for +1923C/T. We used a different method depending on the condition: OR1 = OR3 = 1 and OR2 = 1, recessive model; OR1 = OR2 = 1 and OR3 = 1, dominant model; OR2 = 1/OR3 = 1 and OR1 = 1, completely over-dominant model; and OR1 > OR2 > 1 and OR1 > OR3 > 1 (or OR1 < OR2 < 1 and OR1 < OR3 < 1), codominant model.^[12]^ Heterogeneity of data was evaluated using the Q statistic and further analyzed by the *I*^2^ test.^[13]^ If *I*^2^ <50%, a fixed-effect model was used^[14]^; otherwise, a random-effect model was used as appropriate.^[15]^ Subgroup analysis was conducted to assess ethnicity, age, and atopic status. A funnel plot was used to verify the potential publication bias by Egger linear regression test. Statistical analyses were conducted using STATA 11.2 (Stata, College Station, TX). A *P* value <.05 was considered statistically significant.

## 3 Results

### 3.1 Study characteristics

**Figure 1:**
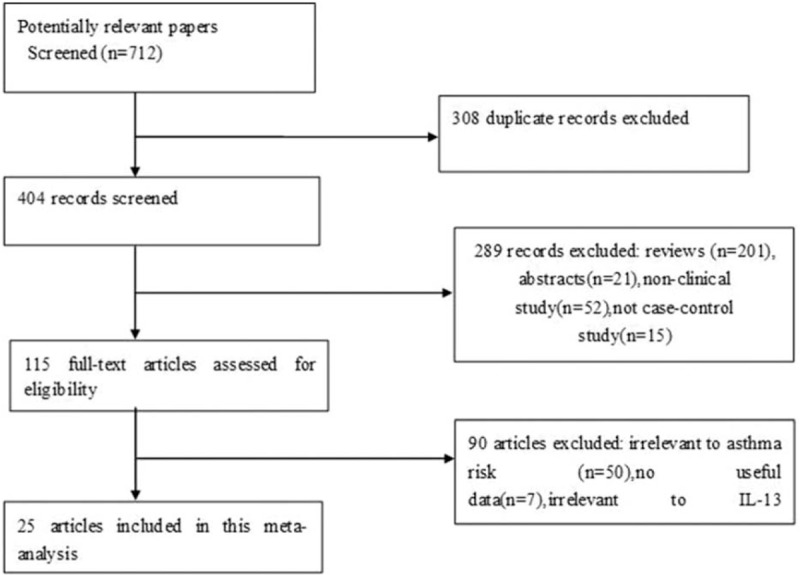
Study flow chart.

The process of selection is shown in Fig. 1. A total of 712 articles were chosen after searching and screening. Ultimately, 25 eligible case-control studies were included, all of which assessed the relationship between IL-13 +2044G/A and +1923C/T polymorphism and asthma risk; the studies represented 16,917 asthmatic cases and 32,522 controls.^[16–40]^ Of these 25 studies, 18 reported IL-13 +2044G/A and 11 reported IL-13 +1923C/T. Eleven studies were conducted on adults, 7 on children, and 4 on both adults and children. Regarding ethnicity among the included studies, 15 studies examined Asians, 9 studies examined Caucasians, and 1 study examined Africans. The major characteristics of each group are in Table 1. Genotype numbers and results of +2044G/A and +1923C/T HWE examination are listed in Tables 2 and 3.

**Table 1:**
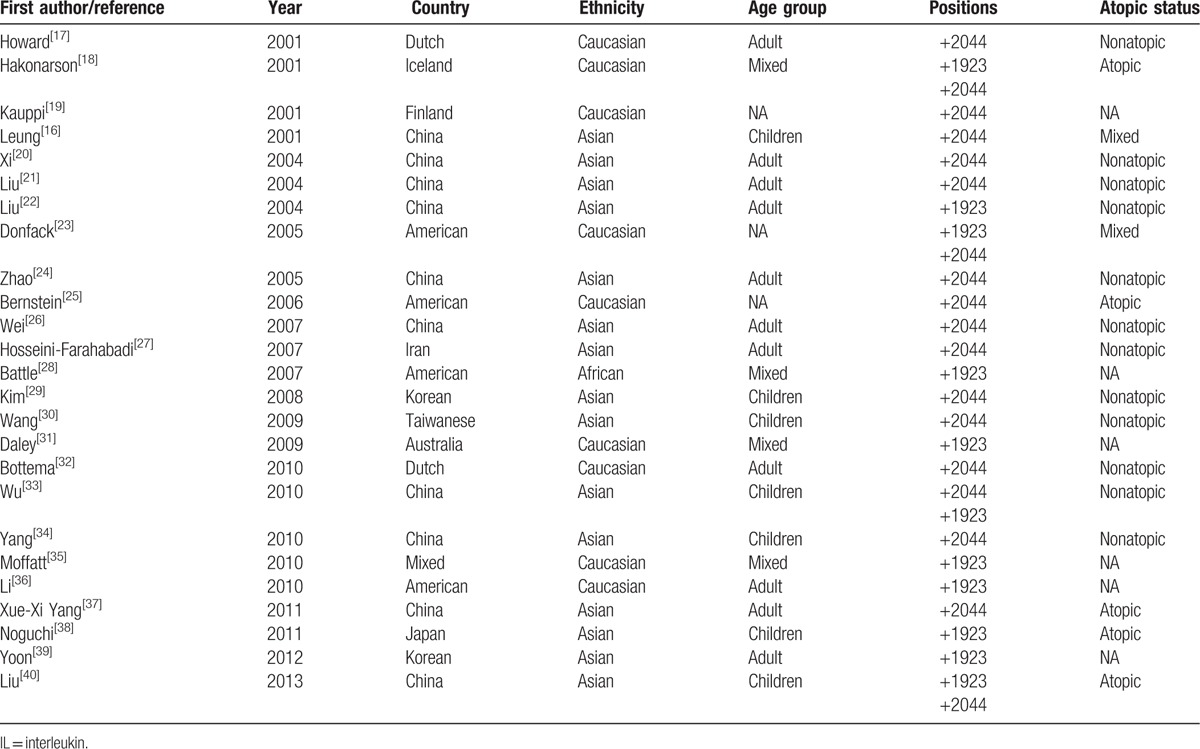
Main characteristics of 25 case-control studies in asthmatic patients showing methods for analyzing IL-13 gene promoter +2044 and +1923 regions.

**Table 2:**
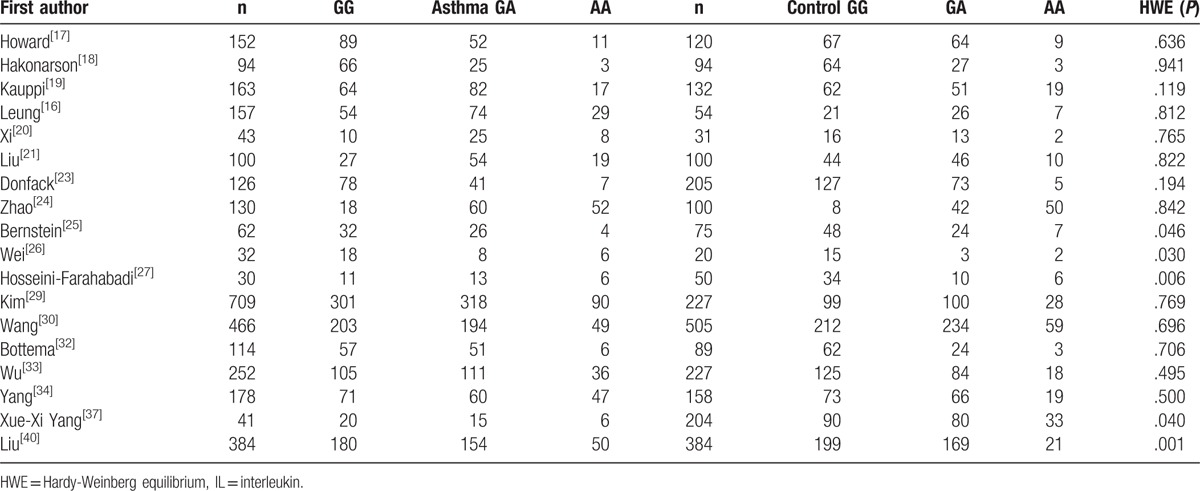
Distribution of IL-13 +2044G/A genotype among patients and controls.

**Table 3:**
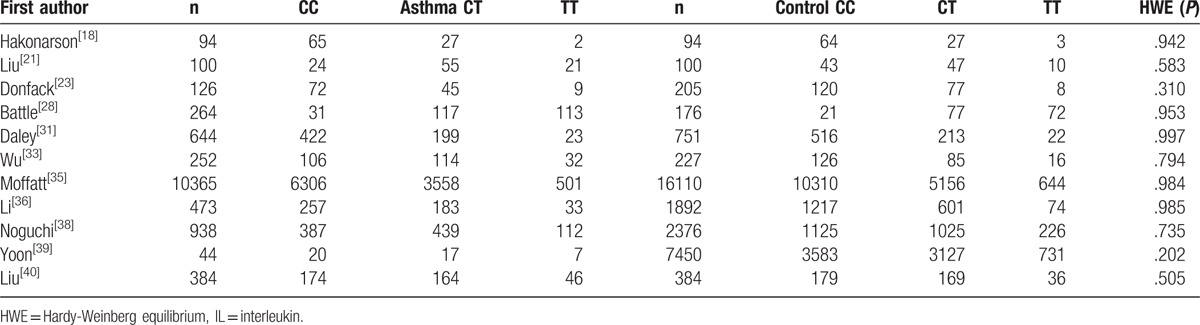
Distribution of IL-13 +1923C/T genotype among patients and controls.

### 3.2 Meta-analysis of available data

#### 3.2.1 IL-13 +2044G/A

**Figure 2:**
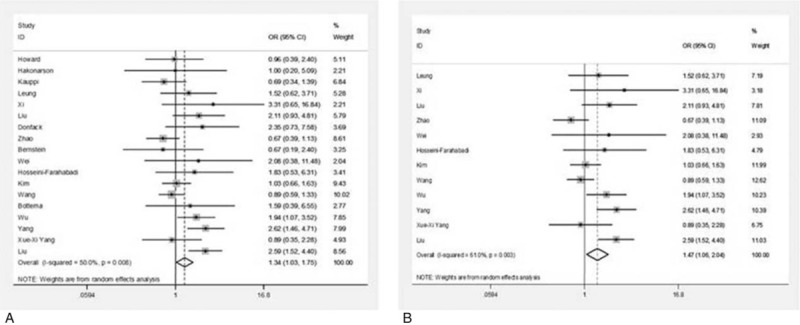
Forest plot of ORs and 95% CIs of +2044G/A between asthmatics and controls in studies related to HWE (A), Asian (B) for AA versus GG + GA test. CI = confidence interval, HWE = Hardy-Weinberg equilibrium, OR = odds ratio.

Table 4 shows results of the meta-analysis for IL-13 +2044G/A and asthma. We analyzed the OR1 (genotype AA vs GG), OR2 (genotype GA vs GG), and OR3 (genotype AA vs GA); we choose the recessive model of analysis (genotypes AA vs GG+GA; OR 1.34, 95% CI 1.03–1.75, *P* = .031), which indicated that IL-13 +2044G/A increased risk of asthma (Fig. 2). To further evaluate the relationship between IL-13 +2044G/A and asthma, we conducted subgroup analyses. IL-13 +2044G/A was significantly associated with Asians (OR 1.47, 95% CI 1.06–2.04, *P* = .020) (Fig. 2). Next, we conducted further analyses on age and atopic status within the Asian subgroups. Increased asthma risk was found in children (OR 1.59, 95% CI 1.05–2.40, *P* = .030), but not adults (OR 1.31, 95% CI 0.75–2.27, *P* = .344), in both atopic (OR 1.63, 95% CI 0.58–4.61, *P* = .355) and in nonatopic asthmatics (OR 1.41, 95% CI 0.97–2.05, *P* = .071).

**Table 4:**
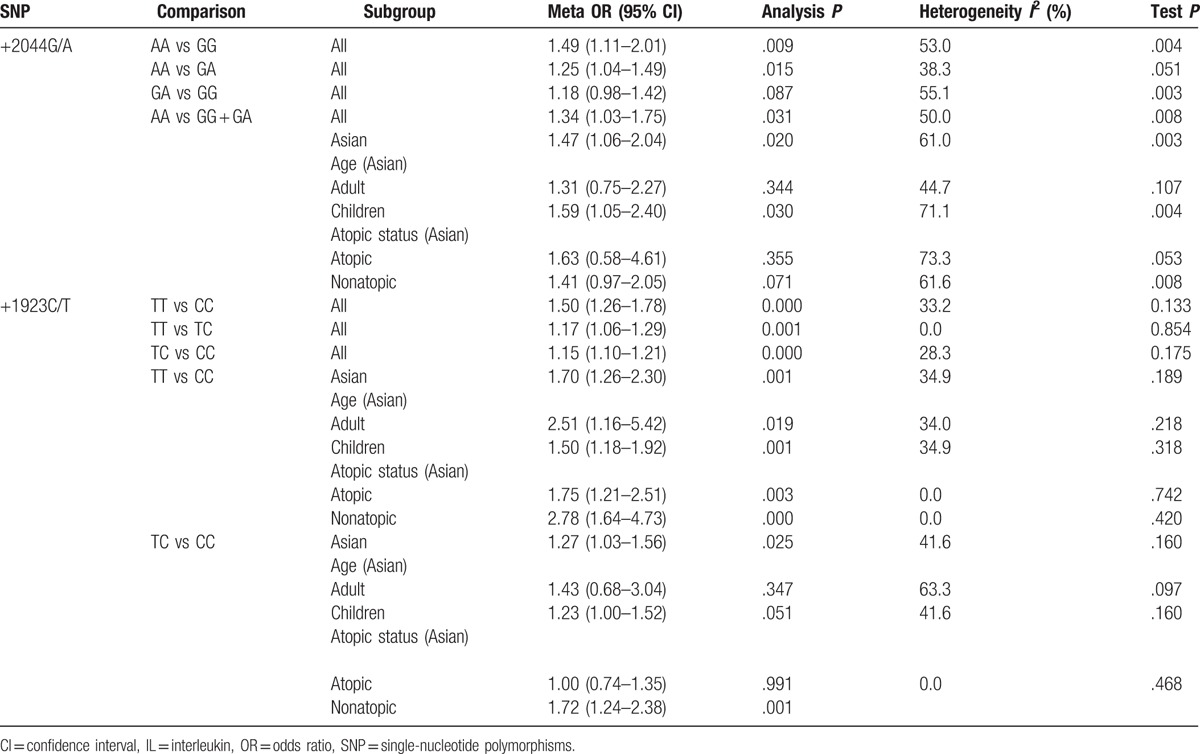
Meta-analysis of the IL-13 polymorphisms in asthma.

In short, the IL-13 +2044G/A polymorphism was significantly associated with asthma. In the subgroup based on Asian ethnicity, there was association between the IL-13 +2044G/A polymorphism and asthma, especially in children, indicating that geographic and age difference may lead to heterogeneity among studies. IL-13 +2044G/A maybe a risk factor in Asian children.

#### 3.2.2 IL-13 +1923C/T

**Figure 3:**
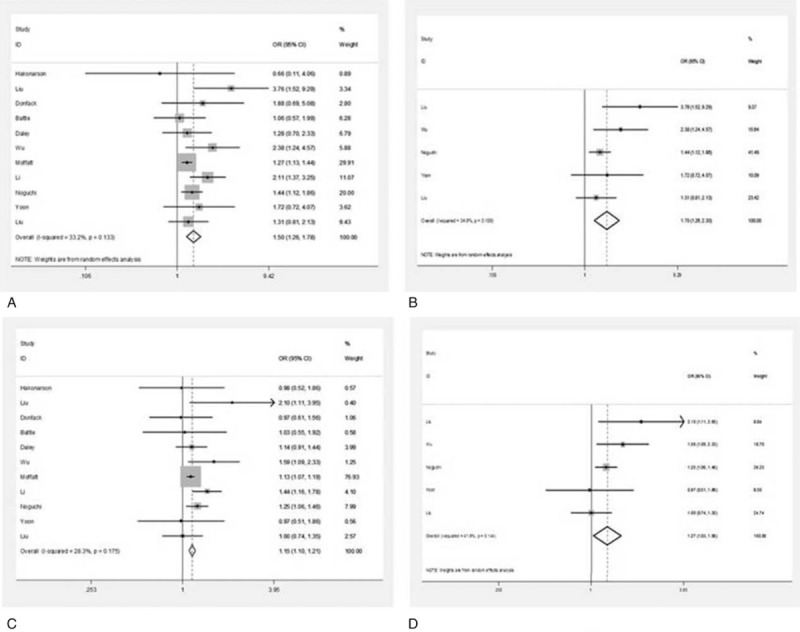
Forest plot of ORs and 95% CIs for +1923C/T between asthmatics and controls in studies including HWE (A), Asian (B) for TT versus CC test; ORs and 95% CIs for +1923C/T between asthmatics and controls in studies including HWE (C), Asian (D) for TC versus CC test. CI = confidence interval, HWE = Hardy-Weinberg equilibrium, OR = odds ratio.

The associations between IL-13 +1923C/T polymorphism and asthma are showed in Table 4. Exploring the comparisons between OR1 (genotype TT vs CC), OR2 (genotype TC vs CC), and OR3 (genotype TT vs TC), we determined that the genetic model was most likely dominant (genotypes TT vs CC OR 1.50, 95% CI 1.26–1.78, *P* = .000; TC vs CC OR 1.15, 95% CI 1.10–1.21, *P* = .000). This model showed that IL-13 +1923C/T increased asthma risk (Fig. 3). A fixed-effect model was chosen for subgroup analysis, on which significant associations were found between IL-13 +1923C/T and Asians (Fig. 3) (OR 1.70,95% CI 1.26–2.30, *P* = .001), including both Asian adults (OR 2.51, 95% CI 1.16–5.42, *P* = .019) and children (OR 1.50, 95% CI 1.18–1.92, *P* = .001), and both atopic (OR 1.75, 95% CI 1.21–2.51, *P* = .003) and nonatopic asthmatics (OR 2.78, 95% CI 1.64–4.73, *P* = .000) for TT versus CC. Similarly, significant associations were also observed among Asians (OR 1.27, 95% CI 1.03–1.56, *P* = .025) for TC versus CC (Fig. 3). However, there was no significant association between Asian age and atopic status for TC versus CC.

In sum, the IL-13 +1923C/T polymorphism was associated with asthma risk in our meta-analysis. IL-13 +1923C/T was associated with asthma among Asians; for TT versus CC, IL-13 +1923C/T associated with Asian adults and children, and with atopic status, indicating that the T allele plays an important role in asthma.

### 3.3 Study heterogeneity and publication bias

**Figure 4:**
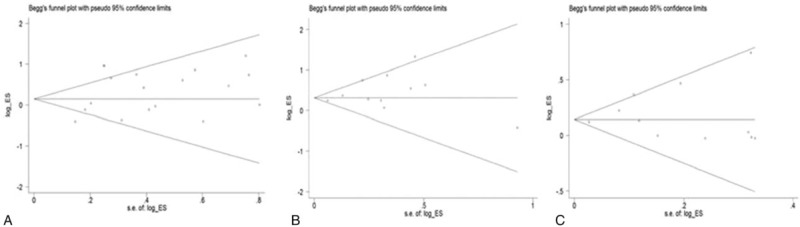
Funnel plot for asthma risk and the IL-13 2044G/A (A) or +1923C/T (B, C) polymorphisms. IL = interleukin.

Sensitivity analyses did not change the initial results. There was little modification of the estimates after exclusion of individual studies. Publication bias in the included literature did not show evidence of remarkable asymmetry (Fig. 4), as supported by Egger test (all *P* > .1).

## 4 Discussion

Asthma is 1 of the most common chronic airway inflammations in humans. Asthma is estimated to cause over 3000 deaths and cost over $56 billion annually in medical expenses.^[41]^ The imbalance of type 1 and type 2 immune responses is the main cause of asthma. Th2 cells mainly secrete cytokines IL-4, IL-5, and IL-13, which stimulate type 2 immunity to induce an immune response.^[42,43]^ IL-13 is an important mediator of asthma exacerbations. Clinical trial data for IL-13-directed therapies show a strong effect on decreasing asthma exacerbation, implicating IL-13 as a mediator of inflammation in asthma.^[44]^ In addition, many experimental results have demonstrated that IL-13 may direct increased airway inflammation and is involved in airway remodeling in asthma. Introduction of exogenous IL-13 into murine airways results in eosinophilic and lymphocytic increases, airway muscle contraction, and airway hyper-responsiveness.^[5,6]^ Furthermore, the inhibition of IL-13 alone in vivo prevents and reverses established mucus cell changes, indicating that IL-13 plays an important role in mucus hyperproduction.^[45,46]^ These cumulative results suggest that IL-13 is the major effector of asthma exacerbation. Moreover, in the Human Genome Project, it was very popular to use SNPs to detect the localization of the genetic determinants of human disease. Numerous studies have revealed that the IL-13 +2044A/G SNP markedly increases the risk of developing asthma.^[16–21,23–27,29,30,32–34,37,40]^ Meanwhile, many studies have found that the IL-13 +1923C/T polymorphism is associated with asthma risk.^[18,22,23,28,31,33,35,36,38–40]^ Thus, it is interesting to study the +2044A/G and +1923C/T polymorphisms associated with asthma.

In this study, we included 25 case-control studies with specific information about the relationship between the +2044A/G and +1923C/T polymorphisms in IL-13 and asthma in a meta-analysis. Although 4 meta-analyses have revealed evidence that these 2 polymorphisms are associated with significantly increased risk of asthma in overall analyses,^[8–11]^ none reported the relationship between these polymorphisms within specific Asian age groups and atopic status. Wang et al^[10]^ did not analyze whether +2044A/G was associated with Asian ethnicity. Our findings are in agreement with linkage studies showing that these 2 polymorphisms are associated with asthma risk factors, especially in Asians. The difference between the other meta-analyses and ours is that we focused specifically on Asian subgroups. The results of our meta-analysis suggest that there is an association between the IL-13 +2044G/A and +1923C/T polymorphisms, and asthma susceptibility. Our subgroup meta-analysis also demonstrates that the IL-13 +2044G/A and +1923C/T polymorphisms are associated with Asian ethnicity; in particular, the IL-13+2044G/A polymorphism is associated with participants who were Asian adults or children, and with Asians who had atopic status. However, the +1923C/T polymorphisms were not associated with Asian age group or atopic status. We also carried out sensitivity analysis to assess the stability of this meta-analysis. Sequential removal of each study did not alter the conclusions regarding increased asthma risk, suggesting that these results are reliable. Thus, there is no publication bias in our meta-analysis.

There are several limitations to our meta-analysis. First, the number of published studies was insufficient for comprehensive analysis, particularly for the +1923C/T polymorphism; there were only 2 studies that included atopic and nonatopic status within Asians for the +1923C/T polymorphisms analysis. Second, heterogeneity may have affected the meta-analysis. Third, because it was not included in these studies’ original data, we were unable to evaluate of the effects of gene-gene and gene-environment interactions within the asthma patient population.

## 5 Conclusions

To the best of our knowledge, this is the first meta-analysis to explain the relationships between the +2044A/G and +1923C/T polymorphisms, and age and atopic status, specifically within Asian samples. Our study results suggest that the IL-13 +2044A/G and +1923C/T polymorphisms are significantly associated with asthma risk; specifically, IL-13 +2044A/G is associated with Asian ethnicity among adults and children with atopic status. Further investigations should also consider gene-gene and gene-environment interactions, and well-designed studies with larger samples should be considered.

## References

[1] Lambrecht BN Hammad H The immunology of asthma. Nature Immunol 2015;16:45–56. 25521684 10.1038/ni.3049

[2] Magnan AO Mely LG Camilla CA Assessment of the Th1/Th2 paradigm in whole blood in atopy and asthma. Increased IFN-gamma-producing CD8(+) T cells in asthma. Am J Respir Crit Care Med 2000;161:1790–6. 10852746 10.1164/ajrccm.161.6.9906130

[3] Barnes PJ IL-10: a key regulator of allergic disease. Clin Exp Allergy 2001;31:667–9. 11422123 10.1046/j.1365-2222.2001.01118.x

[4] Brusselle GG Kips JC Tavernier JH Attenuation of allergic airway inflammation in IL-4 deficient mice. Clin Exp Allergy 1994;24:73–80. 8156448 10.1111/j.1365-2222.1994.tb00920.x

[5] Wills-Karp M Luyimbazi J Xu X Interleukin-13: central mediator of allergic asthma. Science 1998;282:2258–61. 9856949 10.1126/science.282.5397.2258

[6] Grunig G Warnock M Wakil AE Requirement for IL-13 independently of IL-4 in experimental asthma. Science 1998;282:2261–3. 9856950 10.1126/science.282.5397.2261 PMC3897229

[7] Heinzmann A Mao XQ Akaiwa M Genetic variants of IL-13 signalling and human asthma and atopy. Human Mol Genet 2000;9:549–59. 10699178 10.1093/hmg/9.4.549

[8] Nie W Liu Y Bian J Effects of polymorphisms −1112C/T and +2044A/G in interleukin-13 gene on asthma risk: a meta-analysis. PloS One 2013;8:e56065. 23437086 10.1371/journal.pone.0056065 PMC3577847

[9] Yang H Dong H Dai Y Association of interleukin-13 C-1112T and G+2044A polymorphisms with asthma: a meta-analysis. Respirology 2011;16:1127–35. 21762457 10.1111/j.1440-1843.2011.02021.x

[10] Wang ZD Lian D Shen JL Association between the interleukin-4, interleukin-13 polymorphisms and asthma: a meta-analysis. Mol Biol Rep 2013;40:1365–76. 23070918 10.1007/s11033-012-2180-0

[11] Liu Y Liu T Nie W Interleukin-13+1923C/T polymorphism is associated with asthma risk: a meta-analysis. Biomed Res Int 2013;2013:394316. 23841068 10.1155/2013/394316 PMC3693103

[12] Thakkinstian A McElduff P D’Este C A method for meta-analysis of molecular association studies. Stat Med 2005;24:1291–306. 15568190 10.1002/sim.2010

[13] Vangel MG Rukhin AL Maximum likelihood analysis for heteroscedastic one-way random effects ANOVA in interlaboratory studies. Biometrics 1999;55:129–36. 11318146 10.1111/j.0006-341x.1999.00129.x

[14] Higgins JP Thompson SG Deeks JJ Measuring inconsistency in meta-analyses. BMJ 2003;327:557–60. 12958120 10.1136/bmj.327.7414.557 PMC192859

[15] DerSimonian R Laird N Meta-analysis in clinical trials. Control Clin Trials 1986;7:177–88. 3802833 10.1016/0197-2456(86)90046-2

[16] Leung TF Tang NL Chan IH A polymorphism in the coding region of interleukin-13 gene is associated with atopy but not asthma in Chinese children. Clin Exp allergy 2001;31:1515–21. 11678850 10.1046/j.1365-2222.2001.01212.x

[17] Howard TD Whittaker PA Zaiman AL Identification and association of polymorphisms in the interleukin-13 gene with asthma and atopy in a Dutch population. Am J Respir Cell Mol Biol 2001;25:377–84. 11588017 10.1165/ajrcmb.25.3.4483

[18] Hakonarson H Bjornsdottir US Ostermann E Allelic frequencies and patterns of single-nucleotide polymorphisms in candidate genes for asthma and atopy in Iceland. Am J Respir Crit Care Med 2001;164:2036–44. 11739132 10.1164/ajrccm.164.11.2101086

[19] Kauppi P Lindblad-Toh K Sevon P A second-generation association study of the 5q31 cytokine gene cluster and the interleukin-4 receptor in asthma. Genomics 2001;77:35–42. 11543630 10.1006/geno.2001.6613

[20] Xi D Pan S Cui T Association between IL-13 gene polymorphism and asthma in Han nationality in Hubei Chinese population. J Huazhong Univ Sci Technolog Med Sci 2004;24:219–22. 15315330 10.1007/BF02831994

[21] Liu J Wu B Chen H Relationships among IL-13 gene polymorphism, asthma and plasma cytokine levels. J Clin Pulm Med 2004;122–4.

[22] Liu J Wu B Chen H Correlation between +1923C/T polymorphism in IL-13 gene and asthma and its impact on total plasma IgE levels. J Guangdong Med Coll 2004;22:211–4.

[23] Donfack J Schneider DH Tan Z Variation in conserved non-coding sequences on chromosome 5q and susceptibility to asthma and atopy. Respir Res 2005;6:145. 16336695 10.1186/1465-9921-6-145 PMC1325232

[24] Kaishu Z Jirong L Shanyu L Correlationship between interleukin-13 genotype and phenotype in children with bronchial asthma. J Clin Pediatr 2005;23:312–4.

[25] Bernstein DI Wang N Campo P Diisocyanate asthma and gene-environment interactions with IL4RA, CD-14, and IL-13 genes. Ann Allergy Asthma Immunol 2006;97:800–6. 17201240 10.1016/S1081-1206(10)60972-6

[26] Ming W Hongchun S Shujuan L Relationship between IL-13 exon 4 gene polymorphisms A2044G and the patients with asthma. Clin Med J China 2007;14:329–30.

[27] Hosseini-Farahabadi S Tavakkol-Afshari J Rafatpanah H Association between the polymorphisms of IL-4 gene promoter (-590C>T), IL-13 coding region (R130Q) and IL-16 gene promoter (-295T>C) and allergic asthma. Iran J Allergy Asthma Immunol 2007;6:9–14. 17303923

[28] Battle NC Choudhry S Tsai HJ Ethnicity-specific gene-gene interaction between IL-13 and IL-4Ralpha among African Americans with asthma. Am J Respir Crit Care Med 2007;175:881–7. 17303794 10.1164/rccm.200607-992OC PMC1899298

[29] Kim HB Kang MJ Lee SY Combined effect of tumour necrosis factor-alpha and interleukin-13 polymorphisms on bronchial hyperresponsiveness in Korean children with asthma. Clin Exp Allergy 2008;38:774–80. 18341619 10.1111/j.1365-2222.2008.02965.x

[30] Wang JY Liou YH Wu YJ An association study of 13 SNPs from seven candidate genes with pediatric asthma and a preliminary study for genetic testing by multiple variants in Taiwanese population. J Clin Immunol 2009;29:205–9. 18931892 10.1007/s10875-008-9256-6

[31] Daley D Lemire M Akhabir L Analyses of associations with asthma in four asthma population samples from Canada and Australia. Hum Genet 2009;125:445–59. 19247692 10.1007/s00439-009-0643-8

[32] Bottema RW Nolte IM Howard TD Interleukin 13 and interleukin 4 receptor-alpha polymorphisms in rhinitis and asthma. Int Arch Allergy Immunol 2010;153:259–67. 20484924 10.1159/000314366

[33] Wu X Li Y Chen Q Association and gene-gene interactions of eight common single-nucleotide polymorphisms with pediatric asthma in middle china. J Asthma 2010;47:238–44. 20394509 10.3109/02770900903509099

[34] Yang L Zhang Y Liu Q Genetic Arg144Gln polymorphism of interleukin-13 and asthma in children. Mod Med J China 2010;12:46–7.

[35] Moffatt MF Gut IG Demenais F A large-scale, consortium-based genomewide association study of asthma. N Engl J Med 2010;363:1211–21. 20860503 10.1056/NEJMoa0906312 PMC4260321

[36] Li X Howard TD Zheng SL Genome-wide association study of asthma identifies RAD50-IL13 and HLA-DR/DQ regions. J Allergy Clin Immunol 2010;125:328–35. e11. 20159242 10.1016/j.jaci.2009.11.018 PMC2824608

[37] Yang XX Li FX Wu YS Association of TGF-beta1, IL-4 and IL-13 gene polymerphisms with asthma in a Chinese population. Asian Pac J Allergy Immunol 2011;29:273–7. 22053598

[38] Noguchi E Sakamoto H Hirota T Genome-wide association study identifies HLA-DP as a susceptibility gene for pediatric asthma in Asian populations. PLoS Genet 2011;7:e1002170. 21814517 10.1371/journal.pgen.1002170 PMC3140987

[39] Yoon D Ban HJ Kim YJ Replication of genome-wide association studies on asthma and allergic diseases in Korean adult population. BMB Rep 2012;45:305–10. 22617455 10.5483/bmbrep.2012.45.5.305

[40] Liu Q Hua L Fang D Interleukin-13 and RANTES polymorphisms in relation to asthma in children of Chinese Han nationality. Asian Pac J Allergy Immunol 2013;31:247–52. 24053708 10.12932/AP0298.31.3.2013

[41] Barnett SB Nurmagambetov TA Costs of asthma in the United States: 2002–2007. J Allergy Clin Immunol 2011;127:145–52. 21211649 10.1016/j.jaci.2010.10.020

[42] Spellberg B Edwards JE Jr Type 1/type 2 immunity in infectious diseases. Clin Infect Dis 2001;32:76–102. 11118387 10.1086/317537

[43] Voehringer D Reese TA Huang X Type 2 immunity is controlled by IL-4/IL-13 expression in hematopoietic non-eosinophil cells of the innate immune system. J Exp Med 2006;203:1435–46. 16702603 10.1084/jem.20052448 PMC2118302

[44] Davoine F Cao M Wu Y Virus-induced eosinophil mediator release requires antigen-presenting and CD4+ T cells. J Allergy Clin Immunol 2008;122:69–77. e1–2. 18472150 10.1016/j.jaci.2008.03.028

[45] Blease K Jakubzick C Westwick J Therapeutic effect of IL-13 immunoneutralization during chronic experimental fungal asthma. J Immunol 2001;166:5219–24. 11290806 10.4049/jimmunol.166.8.5219

[46] McKenzie GJ Emson CL Bell SE Impaired development of Th2 cells in IL-13-deficient mice. Immunity 1998;9:423–32. 9768762 10.1016/s1074-7613(00)80625-1

